# Identification of Proteins of Altered Abundance in Oil Palm Infected with *Ganoderma boninense*

**DOI:** 10.3390/ijms15035175

**Published:** 2014-03-24

**Authors:** Jameel R. Al-Obaidi, Yusmin Mohd-Yusuf, Nurhanani Razali, Jaime Jacqueline Jayapalan, Chin-Chong Tey, Normahnani Md-Noh, Sarni Mat Junit, Rofina Yasmin Othman, Onn Haji Hashim

**Affiliations:** 1 Agro-Biotechnology Malaysia Institutes, c/o MARDI Headquarters, Serdang 43400, Selangor, Malaysia; E-Mail: jr_alobaidi@yahoo.com; 2 Centre for Foundation Studies in Science, University of Malaya, Kuala Lumpur 50603, Malaysia; E-Mail: yusmin_y@um.edu.my; 3 Centre for Research in Biotechnology for Agriculture, University of Malaya, Kuala Lumpur 50603, Malaysia; E-Mail: yasmin@um.edu.my; 4 Department of Molecular Medicine, Faculty of Medicine, University of Malaya, Kuala Lumpur 50603, Malaysia; E-Mails: hanani@um.edu.my (N.R.); sarni@um.edu.my (S.M.J.); 5 University of Malaya Centre for Proteomics Research (UMCPR), University of Malaya, Kuala Lumpur 50603, Malaysia; E-Mail: jaime_jacklyn@um.edu.my; 6 Plant Protection Unit, Sime Darby Sdn Bhd, Km 10, Jalan Banting Kelanang, P.O. Box 207, Banting 42100, Selangor, Malaysia; E-Mails: tchinchong@yahoo.com (C.-C.T.); nanniiez82@gmail.com (N.M.-N.); 7 Institute of Biological Sciences, Faculty of Science, University of Malaya, Kuala Lumpur 50603, Malaysia

**Keywords:** basal stem rot, *Ganoderma boninense*, biomarker, oil palm, two-dimensional gel electrophoresis, ESI-TRAP

## Abstract

Basal stem rot is a common disease that affects oil palm, causing loss of yield and finally killing the trees. The disease, caused by fungus *Ganoderma boninense*, devastates thousands of hectares of oil palm plantings in Southeast Asia every year. In the present study, root proteins of healthy oil palm seedlings, and those infected with *G. boninense*, were analyzed by 2-dimensional gel electrophoresis (2-DE). When the 2-DE profiles were analyzed for proteins, which exhibit consistent significant change of abundance upon infection with *G. boninense*, 21 passed our screening criteria. Subsequent analyses by mass spectrometry and database search identified caffeoyl-CoA *O*-methyltransferase, caffeic acid *O*-methyltransferase, enolase, fructokinase, cysteine synthase, malate dehydrogenase, and ATP synthase as among proteins of which abundances were markedly altered.

## 1. Introduction

Palm oil, which accounts for nearly half of the edible oil worldwide, is largely derived from mesocarp of the fruit of the African oil palm, *Elaeis guineensis* Jacq. Unfortunately, the oil palm plantations in Southeast Asia, especially Malaysia and Indonesia, are threatened by basal stem rot (BSR) infection. BSR can cause up to 50% loss of palm plantations [1,2]. The disease is caused by the basidiomycetes fungus, *Ganoderma boninense*, which is most damaging to the oil palm. Currently available control methods for BSR disease, such as mechanical and chemical treatment, as well as cultural practices, have been proven unsatisfactory due to the fact that *G. boninense* has various resting stages, such as melanised mycelium, basidiospores, and pseudosclerotia [3].

Many studies have documented changes in expression of proteins of plants during an infection. Examples include alteration of the rice proteome after being infected by the rice blast fungus *Magnaporthe grisea* [4], identification of *Medicago truncatula* proteins induced in roots after infection with the pathogenic oomycete *Aphanomyces euteiches* [5], interaction between the pathogenic fungus *Fusarium graminearum* and wheat [6], and study of proteomic response of date palm to the endophytic colonization by the entomopathogenic fungi *Beauveria bassiana*, *Lecanicillium dimorphum*, and *L. cf. psalliotae* [7]. These studies have led to the discovery of proteins involved in the plant defense response and also improved understanding of why other plants are also susceptible [8]. In addition, evidences to suggest differences in susceptibility to BSR disease between germplasm materials from different genetic origins have been generated [9]. This provides hope in generating oil palm varieties with reduced levels of susceptibility using existing genetic materials. At the same time, there is also interest in developing early diagnostic tools for detection of the pathogen in oil palms so that intervention may be promptly initiated to avoid losses [10,11].

In recent years, the application of proteomic approaches as a tool for protein expression analysis and identification has been highly efficient. Proteomic plays an increasingly important role in addressing protein expression issues and has become an approach which is necessary and complementary in the post-genomic era. Considerable reports have described the use of proteomic in the isolation of proteins for agronomic applications [4,5,7,12,13]. However, relatively little information is available regarding the biochemical and physiological interactions of proteins in the plant system, particularly the oil palm, during an infection [14].

Plants usually respond to fungal infection by eliciting different mechanisms, including defense [15]. For plant–pathogen interaction studies, comparative proteomics proves to be one of the best approach as it has emerged as a promising tool for global analyses of protein expression levels in the recent past [16]. Although genomics-based investigation of host-pathogen interactions can provide valuable information on the changes in gene expression, especially with the recently reported oil palm genome [17], the investigation into changes in protein abundance is also important, in order to identify those proteins that are essential during such interactions.

In view of this, as well as the need to develop diagnostic tools for early detection of the *G. boninense* infection in oil palms, the present study was carried out to investigate alteration of abundance of root proteins in response to a progressive basal stem rot infection caused by *G. boninense* using the gel-based proteomic approach.

## 2. Results

### 2.1. Analysis of Oil Palm Root Proteins by 2-DE

**Figure 1.**
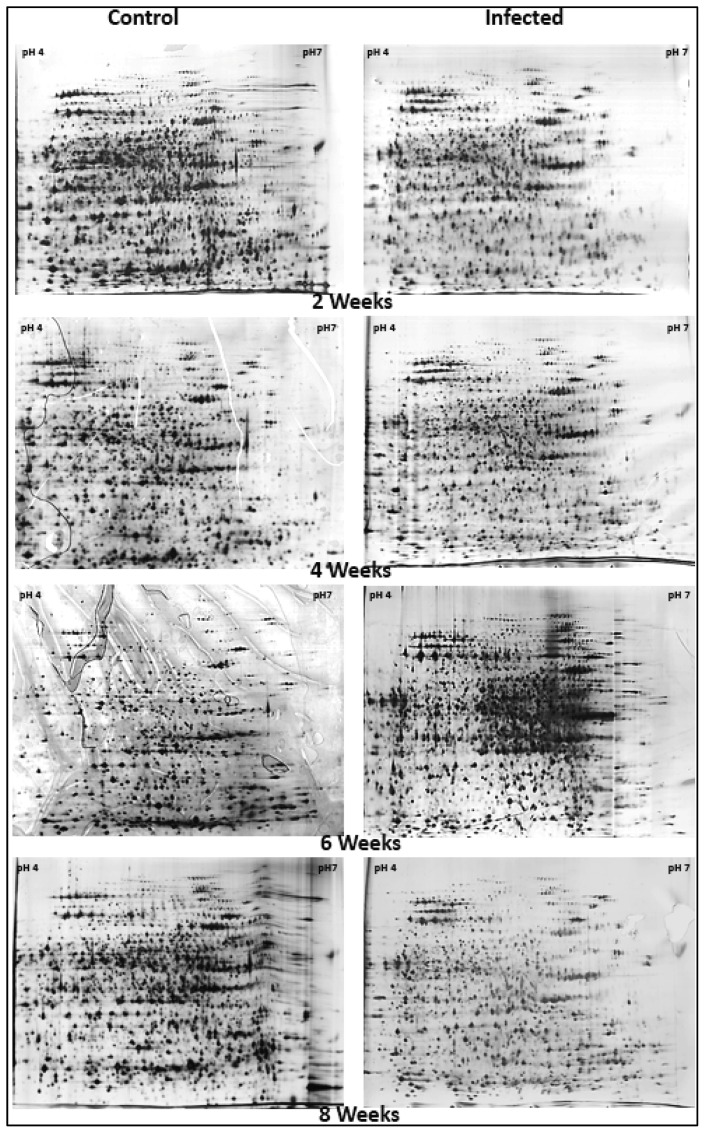
Representative 2-DE profiles of control and infected oil palm root proteins. Root proteins from control and infected oil palm tissues were harvested at two, four, six, and eight weeks before being subjected to the 2-DE profiling. Acid side of 2-DE gels is to the left and relative molecular mass declines from the top.

To investigate alteration of root protein abundance in response to progressive BSR infection caused by *G. boninense*, 2-DE root protein profiles of *E. guineensis* at different timeframes of harvest were analyzed. Triplicate gels were obtained from three independent experiments and the representative gels for each of the infected sample, as well as their respective controls, are shown in Figure 1.

### 2.2. Image Analysis of 2-DE Oil Palm Root Protein Profiles

To avoid generating excessive information with a possible false positive data, stringent comparison was opted. The relative protein spot intensities of different gels were averaged during image analysis. Spots that showed inconsistent intensity among extracts of the same harvest timeframe, *i.e.*, not present in all gel images compared, were excluded from the study. Our results showed that there were 2411 consistent protein spots depicted on the triplicate gels after two weeks of inoculation, while 2342, 2289 and 2044 spots were consistently detected in all three gels after four, six, and eight weeks inoculation, respectively. The control gels showed detection of 2289, 2719, 2294, and 2419 consistent spots in the gels after two, four, six, and eight weeks inoculation, respectively.

**Figure 2.**
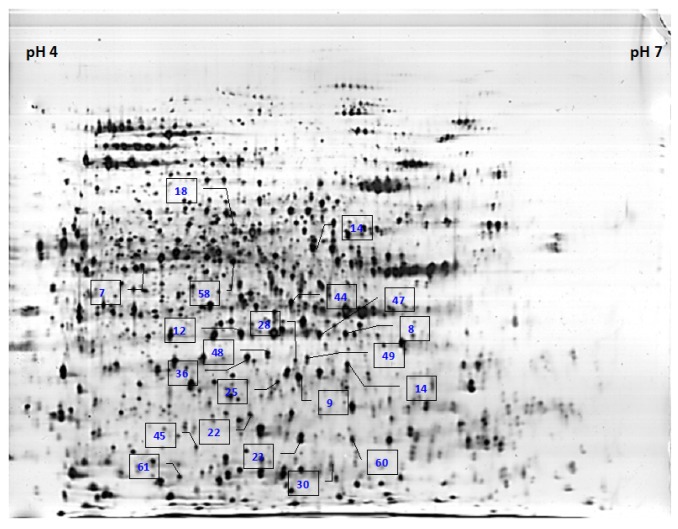
A representative 2-DE profile of control oil palm root proteins harvested at two weeks. Twenty-one proteins whose abundances were significantly altered are labeled with their protein ID numbers in the gel.

**Figure 3.**
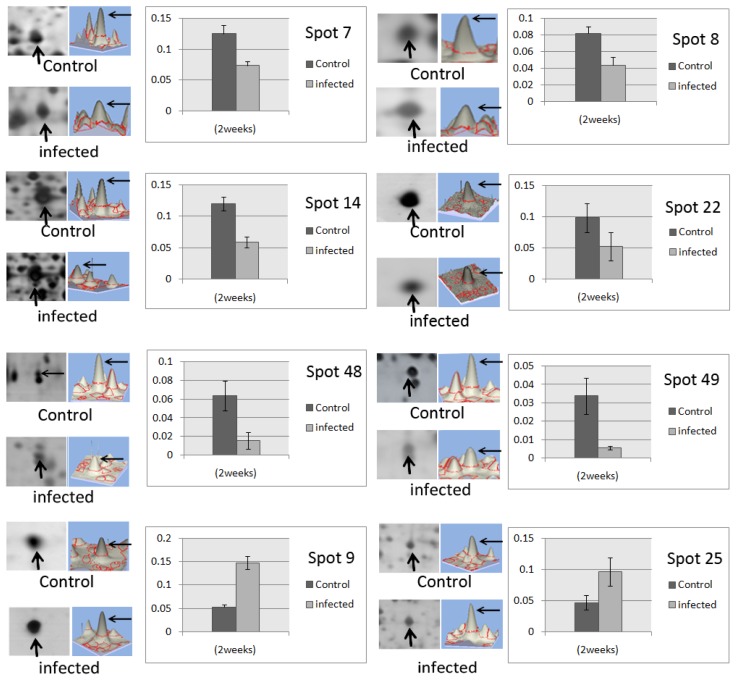
Changes in abundance of oil palm proteins harvested at two weeks after *G. boninense* infection. Histograms demonstrate mean % volume contribution of protein spot. Error bars are standard deviation of the average spot densities.

When image analysis was performed on individual proteins that were detected in the 2-DE gels, 61 spots of altered abundance were detected between control and infected samples throughout the selected timeframes of the study. The mean percentage of volume of the protein spots was analyzed using Imagemaster 2-DE platinum software version 7.0 and the Student’s *t* test was employed to determine protein spots that showed significant change in their abundances (*p* < 0.05). Among the 61 protein spots detected for altered abundance, 21 showed fold changes of >1.5 for at least two timeframes (Figure 2). These 21 proteins showed consistent trend of altered abundance at the different time points of analysis compared to the controls. However, their abundances at the different progressive timeframes after infection were not significantly altered. Figure 3 demonstrates eight of the protein spots of altered abundance, which were subsequently identified with known functions.

### 2.3. Analysis by Tandem Mass Spectrometry

Tandem mass spectrometry (MS/MS) and database query were performed on the 21 root proteins that showed significant altered abundance. Most search queries included 20 to 60 amino acids, resulting from two to six peptide sequences. Using these queries, the homology-based search algorithms yielded identifications with scores significantly better than threshold values. Using this approach, we were able to obtain 10 hits out of the 21 protein spots that were subjected to the MS/MS analysis (Table 1). Among the 10 protein spots, six were metabolic enzymes, *i.e.*, cysteine synthase (spot 9), enolase (spots 14 and 22), malate dehydrogenase (MDH; spot 25), fructokinase 1 (spot 49) and fructokinase 2 (spot 48), and one directly involved in energy generation (spot 7; α subunit of ATP synthase). One of the proteins, *i.e.*, catechol *O*-methyl-transferase (COMT; spot 23) is involved in defense, while two others (spots 28 and 36) were without any known functions [18].

### 2.4. Pathway Interactions and Biological Process Analysis

Subjecting the seven abundance changed proteins to analysis by IPA identified “Gluconeogenesis” as the canonical pathway with highest predicted potential/significance of being affected by altered levels of enolase and malate dehydrogenase (*p* < 4.36 × 10^−8^; Table 2). Two top linking networks were also identified (Table 2). Among these networks, “Cellular Function and Maintenance, Energy Production and Lipid Metabolism” links all seven proteins with other interactomes and generated a score of 20. A score of 2 or higher indicates confidence of at least 99% of not being generated by random chance and higher scores indicate greater confidence. The second linking network identified by IPA was “Amino Acid Metabolism, Small Molecule Biochemistry, and Molecular Transport”, with a score of 2.

**Figure 4.**
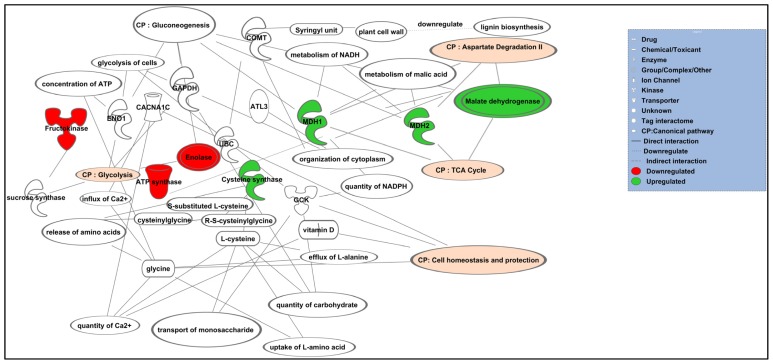
Predicted canonical pathway of altered abundance root proteins of oil palm following infection of *G. boninense*. Lines between proteins represent known interactions. Nodes in red indicate up-regulated proteins while those in green represent down-regulated proteins. Various shapes of nodes represent functional class of proteins. Different arrow shapes represent different types of interactions. Edges are displayed with various labels that describe nature of relationship between the nodes. Abbreviation: CP, Canonical pathway; COMT, Catechol *O*-methyltransferase; MDH, malate dehydrogenase.

Figure 4 shows a graphical representation of the predicted molecular relationships between the two top networks. MDH, enolase, fructokinase, ATP synthase alpha subunit, and COMT were apparently interacted via gluconeogenesis, glycolysis, and homeostasis of cells. On the other hand, cysteine synthase appears to be involved in amino acid metabolism.

## 3. Discussion

A number of proteomic studies have been carried out in order to understand plant response or plant–fungus interactions [17]. The goal of this study was to identify major oil palm root proteins that were altered in abundance after being infected by *G. boninense*. Such proteins may function in the signaling pathways of root tissues or play a role in physiological changes and/or metabolic switch that is a key for plant defense. When biological sample replicates of infected and non-infected roots of oil palm were screened for proteins which exhibit significant but consistent change in abundance, 21 passed our screening criteria. Subjecting the spots to mass spectrometry and database search identified 10 proteins, which included MDH, cysteine synthase, enolase (two distinct protein spots), fructokinase 1 and 2, ATP synthase, and COMT. While the first two enzymes showed higher abundance, the latter were reduced in abundance after being infected by *G. boninense*. The other two identified proteins were without any known function.

In a similar 2-DE-based proteomic study of the oil palm root infected with *G. boninense*, Syahanim *et al.* recently reported the altered abundance of beta-1,3-glucanase (2 spots), early flowering protein 1 (5 spots), gluthathione S-transferase, nucleoside diphosphate kinase (2 spots), thioredoxin H2 and ferritin [19]. However, none of the proteins detected matches those that were identified in the present study. This may be due to the difference of age of the seedlings used, as well as the timeframes of analysis that was carried out. While twelve-month old seedlings were used in the earlier reported work, our study was performed on seedlings that were germinated for one month. The proteins of altered abundance were detected after one week of infection in the earlier study, while our analysis was performed at two weeks post-infection.

MDH has been earlier shown to be elevated in barley and barrel clover leaves during infection with pathogenic fungi, *Fusarium graminearum* and *Uromyces striatus*, respectively [20,21]. Similar results were obtained from the proteomics analysis of *Arabidopsis thaliana* subsequent to an infestation by diamond black moth, *Plutella xylostella* [22]. The data of our study when taken together with those of the earlier reports suggest that MDH appears to be up-regulated when plants were subjected to stress conditions. MDH is the main enzyme involved in the synthesis of malate in the Kreb’s cycle. The enzyme has been shown to be present in plant cell organelles and wall and also reported to have an essential role in removing toxic radicals formed during oxidative stress [23]. Over-expression of MDH may be an indication of metabolic disorder in the oil palm tissues.

Cysteine synthase is a pyridoxal 5-phosphate-dependent enzyme that catalyzes a variety of reactions, including decarboxylation, transamination, deamination, and *trans*-sulfuration in the metabolism of amino acids [24]. In plants, this enzyme plays an important role in biosynthesis of tetrapyrrole structures that make chlorophyll [25,26]. Higher abundance of cysteine synthase has been shown in the oil palm roots infected with *G. boninense* in this study, and this was similarly observed in the roots of *Malusxiao jinensis* during iron deficiency. As with MDH, higher abundance of cysteine synthase may be a reflection of stress-induced response.

Unlike MDH and cysteine synthase, two enolases were detected in the root tissues of oil palm infected with *G. boninense* and both were reduced in abundance. This is compatible with the results of a previous proteomic study which showed suppression of enolase in tomatoes infected with *Ralstonia solanacearum* [27]. Enolase is involved in the metabolism of carbohydrates and is critical for generation of energy during root growth [28]. Therefore, the lower abundance of enolase after *G. boninense* infection may be due to limited supply of sugars in infected oil palm roots [27]. However, enolase transcripts had been shown to be induced in tomatoes that were invaded by *Oidium lycopersicum* [29] and activity of the enzyme was also induced during *Agrobacterium tumefaciens–Beta vulgaris* interaction [30]. As the catalytic action of enolase is reversible this is not really surprising. In starch accumulating seeds, such as maize, enolase is also considered part of the gluconeogenesis enzyme [31,32].

Fructokinases 1 and 2 that were shown to be lower in abundance in the oil palm root tissues infected by *G. boninense* play an important role in the metabolism of fructose [33,34]. Decrease of both fructokinases after an infection with *G. boninense* reflects inhibition of fructose metabolism in the oil palm root, just like enolase in glycolysis [33]. Previously, wheat fructokinase had also been shown to be down-regulated during a drought stress. However, the expression of fructokinase was apparently up-regulated in tomatoes during a *Ralstonia solanacearum* infection [29]. Rice fructokinase also showed elevated levels of expression during infection with the fungus, *Magnapor theoryzae* [35].

ATP synthase is an enzyme involved in the synthesis of ATP from ADP via photosynthesis [36]. The alpha subunit of this enzyme was found to be lower in abundance in *G. boninense* infected oil palm root compared to the controls. Reduced levels of mitochondrial ATP synthase gradually decrease the ATP/ADP ratio in the plant cell and alter respiration. This leads to massive induction of alternative respiratory pathways, which restricts growth of the oil palm cells. However, ATP synthase has been found to be overexpressed in rice that had fungal infection [37], as well as chili (*Capsicum annuum*) that was infected with fungi *Fusarium oxysporum* [38]. This is probably part of an early defense response in the plants, which requires more energy.

COMT that was found to be reduced in abundance in the present study is involved in the biosynthesis of lignin [39]. In plants, lignin belongs to a copious class of plant chemicals that play an important role in a range of defense responses [40]. It provides mechanical strength and act as a physical wall for protection of neighboring tissues from further damage [41–43]. A number of studies have reported degradation of lignin during *G. boninense* infection of oil palm [42,44,45]. However, the mechanism underlying the degradation was not clearly explained. Plant COMT has a particularly important role in the synthesis of guaiacyl lignin subunits and in the supply of components for the synthesis of syringyl lignin units [46,47]. Previous studies in tobacco have shown that the suppression of COMT gene caused altered amounts and composition of lignin [48]. Down-regulation of COMT in maize was also reported to cause an obvious decrease in the syringyl unit content and consequentially affect the synthesis of lignin [49].

*G. boninense* belong to the phylum basidiomycetes, which are the only microorganisms with the capability of degrading lignin [50]. Basidiomycetes has been proposed to have special metabolic systems with the ability to degrade a variety of aromatic compounds [51], which are thought to play an important role in the structure and function of the plant cell wall [52]. The induction of the lignification process in plant cells is considered part of a basic host defense response to fungal infection [53]. Hence, the significant reduced abundance of oil palm root COMT observed in our study suggests a reduction of the biosynthesis of lignin, indicating a compromised defense mechanism.

Ingenuity Pathways Analysis (IPA) generated a summary of putative biological effects of *G. boninense* on the root proteins of oil palm. IPA is a widely-used bioinformatics tool to identify biological mechanisms, pathways and functions most relevant to previous experimental datasets of proteins of interest [54]. When the proteins of altered abundance were subjected to analysis using IPA, the eight oil palm root proteins with known functions that were altered in abundance after infection with *G. boninense* appeared to be interconnected via metabolic and defense pathways. MDH, enolase, fructokinase, ATP synthase, and COMT were apparently interacted via gluconeogenesis, glycolysis, and homeostasis of cells, while cysteine synthase appears to be involved in amino acid metabolism. The IPA analysis also provided information on how the root proteins possibly interacted with each other in defense-related metabolism in oil palm. *G. boninense* infection may have induced catabolism in the plant. This is reflected by up-regulation of MDH and down-regulation of enolase, fructokinase, and ATP synthase. Lower abundance of COMT, which are involved in the synthesis of lignin in the oil palm roots, may possibly indicate severity of infection.

## 4. Experimental Section

### 4.1. Plant Materials

Normal uninfected *Dura* x *Pisifera* seedlings of *UR 706/532* x *UR 1679/147* crosses were supplied by Planting Materials Unit of Sime Darby Seeds and Agricultural Services (Banting, Malaysia), following normal nursery practices and certified as *Ganoderma*-free. Plant roots were briefly washed under running tap water, dried, and rapidly frozen in liquid nitrogen.

### 4.2. Ganoderma Culture

Cultures of *G. boninense* PER71 were obtained from *Ganoderma* & Diseases Research of Oil Palm Unit Laboratory, Malaysian Palm Oil Board (MPOB, Kajang, Malaysia). The culture was maintained at the Plant Protection Unit, Sime Darby Research Centre (Banting, Selangor, Malaysia). To fulfill Koch’s Postulates, re-isolation of fungi was carried out from inoculated tissues by cutting discolored roots and stems into 1 cm sections and plated onto *Ganoderma* Selective Medium [55]. Plates were incubated at 25 °C for 2–3 days and observed for formation of brown halos and growth of mycelia from the root and stem sections. Colonies of *Ganoderma* were white on the surface and heavily pigmented on the reverse side. *Ganoderma* isolate culture had an undulating surface in the darkened regions that buckled the agar [56].

### 4.3. Preparation of Wood Blocks for Artificial Inoculation

The rubber wood block (RWB) method for pathogenicity test was modified from that used by Idris *et al.* [57]. RWBs measuring 6 × 6 × 6 cm were used as substrates to cultivate inocula. All RWBs were washed and dried in an oven at 80 °C overnight before autoclaving at 121 °C for 1 h. Each RWB was placed in a plastic bag containing 120 mL of malt extract broth. Bags were sealed, autoclaved at 121 °C for 15 min and left to solidify overnight. The 7-day-old dikaryon inocula on PDA plate were cut into 4 parts and half of an agar block was used to inoculate the RWB. The inoculated RWBs were incubated at 25–28 °C at 60%–70% relative humidity for about 150 days during which time the *G. boninense* mycelia completely covered the wood blocks.

### 4.4. Plant Challenge Experiments

Seedlings, germinated for one month, were artificially inoculated according to a standardized protocol that was earlier reported [58]. Analysis was initiated at 2 weeks post-infection. This was based on our earlier observation on the significant changes of gene expression after two weeks of infection [10].

### 4.5. Protein Extraction

Approximately 200 mg of root tissue was ground using mortar and pestle in the presence of liquid nitrogen. Fine powder was dissolved in 5 mL Tris–buffered phenol (pH 8.8) and 5 mL extraction buffer consisting of 1 M Tris–HCl pH 8.8, 10 mM EDTA, 0.4% β-mercaptoethanol and 0.9% Sucrose. The mixture was centrifuged at 5000× *g* for 10 min. The phenol phase was removed and the aqueous phase was back-extracted with phenol and extraction buffer in a 1:1 ratio. Total proteins in the phenol phase were precipitated with 5 volumes of ice-cold 0.1 M ammonium acetate in 100% methanol. The mixture was kept at −20 °C for 16 h and centrifuged at 5000× *g* for 10 min at 4 °C. Pellet was washed twice in 20 mL mixture of 0.1 M ammonium acetate in 100% methanol, rinsed twice with ice-cold 80% acetone and 70% ethanol. The protein pellet was dried for 5 min and dissolved in 1 mL solubilization buffer containing 7 M urea, 2 M thiourea, 4% CHAPS, and 0.002% of 1% bromophenol blue [59].

### 4.6. Estimation of Proteins Content

Briefly, bovine serum albumin standards were prepared in six dilutions between 0–1.4 mg/mL. Protein solutions were assayed in duplicate. A standard 250 μL of Bradford reagent was added into wells (96 well plate) containing 5 μL of the standard solution or unknown samples. The mixture was incubated at room temperature for at least 5 min but not more than 1 h. The absorbance was measured at 595 nm using Tecan GENios Microplate Reader (Tecan Group Ltd., Männedorf, Switzerland). Concentrations of proteins were estimated by comparison with a standard curve.

### 4.7. 2-DE

Isoelectricfocusing (IEF) was carried out using 24-cm Immobiline dry strips with a linear pH gradient of 3–10 and Multiphor II flatbed electrophoresis system (GE Healthcare, Uppsala, Sweden). The strips were initially rehydrated for 12 h in presence of 200 μg protein samples. IEF was performed at 18 °C in a stepwise manner: 500 V (0.01 h), 3.0 kV (1.5 h), and finally 3.5 kV (17.20 h). Focused strips were equilibrated for 15 min in buffer containing 50 mM Tris–HCl pH 8.8, 6 M urea, 30% (*v*/*v*) glycerol, 2% (*w*/*v*) SDS, and 1% (*w*/*v*) dithiothreitol (DTT), followed by a further 15 min in the same equilibration buffer containing 2.5% (*w*/*v*) iodoacetamide instead of DTT. Electrophoresis was conducted after transferring the strips onto vertical 12% SDS-PAGE gels, at 16 °C using an Ettan DALT-six System (GE Healthcare, Uppsala, Sweden). Separation was carried out in running buffer (25 mM Tris–HCl, 192 mM glycine, 0.1% (*w*/*v*) SDS) at 2 W/gel (0.30 h) and 17 W/gel (5.00 h). Resolved proteins on gels were visualized by silver staining [60].

### 4.8. Gel Scanning and Image Analysis of Protein Spots

Stained gels were scanned using ImageScanner™ III. Analysis of protein spots was performed using ImageMaster 2D Platinum software according to the procedure proposed by the manufacturer (GE Healthcare, Uppsala, Sweden). Abundance of proteins was analyzed in terms of the percentage of volume contribution. Data expressed in this manner is independent of possible errors that may occur due to minute variation in protein loading. Automatic spot detection was performed with default cut-off parameters setting: Smooth—2; Saliency—1; Min area—5.

### 4.9. Mass Spectrometry and Database Query

Samples were prepared for mass spectrometry according to Bringans *et al.* [61]. In brief, protein spots with fold changes of >1.5 for at least two timeframes were excised from gels. The spots were handpicked from all three replicate gels. Proteins were destained with 25 mM NH_4_HCO_3_ in 50% acetonitrile (ACN), dehydrated in absolute ACN and dried by speed vacuum centrifugation. Dried gel was treated with trypsin (12.5 mg/mL) in 25 mM NH_4_HCO_3_ in 10% ACN at 37 °C overnight. Digested peptides were extracted using ACN containing 1% TFA. All peptide solutions of the same protein were pooled, dried and stored at 4 °C until analysis by mass spectrometry. Peptides were analyzed by electrospray ionization mass spectrometry using the Ultimate 3000 nano HPLC system (Dionex, Thermo Fishers Scientific Co., Rockford, IL, USA) coupled to a 4000 Q TRAP mass spectrometer (Applied Biosystems, Thermo Fisher Scientific Co., Foster City, CA, USA) at Proteomics International Pty Ltd. (Nedlands, Australia). Tryptic peptides were loaded onto a C18 PepMap 100, 3 μm, and separated with a linear gradient of water/ACN/0.1% formic acid (*v*/*v*). Data analysis was performed using MASCOT peptide sequence matching software (Matrix Science Ltd., London, UK) against taxonomy *viridiplantae*—green plants entries (containing 1,281,432 sequences) in the Ludwig NR database (Last update: April, 2013, containing 21,306,855 sequences). Search was performed using the following parameters: Fixed modification included was carbamidomethylation of cysteine whilst variable modification included was oxidation of methionine; peptide tolerance was ±0.2 Da, MS/MS tolerance was ±0.2 Da, peptide charge +1, +2, +3 and only monoisotopic mass were included in the search. Trypsin was set as proteolytic enzyme with 1 allowed missed cleavage per peptide. For a confident ID, only proteins with a minimum of 2-matched peptides and MASCOT scores greater than 44 or extensive homology (*p* < 0.05) were considered.

### 4.10. Functional Analysis

The proteomics data were further analyzed using the Ingenuity Pathways Analysis (IPA) software (Ingenuity^®^ Systems, Redwood City, CA, USA) to predict networks that are affected by the change of abundance proteins in response to *G. boninense* infection. Details of identified proteins, their quantitative expression values and *p*-values were imported into the IPA software. Each protein identifier was mapped to its corresponding protein object and was overlaid onto a global molecular network developed from information contained in the Ingenuity Knowledge Base. Network of proteins was then algorithmically generated based on their connectivity. The *p*-value, which indicates probability of biological function assigned to the network was due to chance, was calculated using the right-tailed Fisher exact test.

## 5. Conclusions

In the present study, 21 proteins of altered abundance were detected when root proteins of healthy oil palm seedlings and those infected with *G. boninense* were analyzed by 2-DE. Analyses by mass spectrometry and database search identified caffeoyl-CoA *O*-methyltransferase, caffeic acid *O*-methyltransferase, enolase, fructokinase, cysteine synthase, malate dehydrogenase and ATP synthase as among proteins whose abundances were markedly altered. These proteins appeared to be interconnected via metabolic and defense pathways.

## Figures and Tables

**Table 1.** List of identified proteins whose abundance were altered when oil palm roots were infected with *G. boninense*.

Spot ID	Accession a	Protein	FC b	*p*-value	MW c	p*I*	Score	MP d	Cov (%) e	Organism	Biological process f	Molecular function f
7	G9JLN6	ATP synthase subunit alpha	−1.64	0.0043	55,602	5.84	387	29	33	*Oryza brachyantha*	ATP hydrolysis coupled proton transport; ATP synthesis coupled proton transport	ATP binding
8	Q9SYR8	Catechol *O*-methyltransferase	−1.80	0.002	40,192	5.57	88	2	6	*Thalictrum tuberosum*		*O*-methyltransferase activity
9	I1R814	Cysteine synthase	+3.17	0.0056	43,774	8.76	63	2	4	*Oryza glaberrima*	cysteine biosynthetic process from serine	cysteine synthase activity; transferase activity
14	B9R9N6	Enolase	−2.01	0.0078	48,149	5.71	199	16	28	*Ricinus communis*	glycolysis	magnesium ion binding; phosphopyruvate hydratase activity
22	B3TLU4	Enolase	−1.88	0.00016	48,127	5.98	80	7	11	*Elaeis guineensis*	glycolysis	magnesium ion binding; phosphopyruvate hydratase activity
25	F6HZK0	Malate dehydrogenase	+2.06	0.0040	39,405	6.67	121	5	18	*Vitis vinifera*	cellular carbohydrate metabolic process; malate metabolic process; tricarboxylic acid cycle	L-malate dehydrogenase activity
28	I1HVU4	Uncharacterized protein	+10.43	0.005	38,996	6.09	66	2	4	*Brachypodiu m distachyon*		nutrient reservoir activity
36	M0YUL2	Uncharacterized protein	−2.60	0.00011	38,971	8.47	105	4	13	*Hordeum vulgare*		
48	Q0J8G4	Fructokinase-2	−4.15	0.0072	35,893	5.02	86	3	9	*Oryza sativa*	starch biosynthestic process	ATP binding; fructokinase activity
49	G1FCF5	Fructokinase	−6.04	0.013	36,460	5.61	72	2	7	*Dimocarpus longan*	D-ribose metabolic process	ribokinase activity

a Uniprot accession number;

b fold change;

c molecular weight;

d number of matched peptide;

e sequence coverage;

f http://www.uniprot.org.

**Table 2.** Summary of ingenuity pathway analysis (IPA).

Top canonical pathway	*p* value	Ratio	List of protein
Gluconeogenesis	4.36 × 10^−8^	2/47 (1.043)	Enolase, Malate dehydrogenase
Cell homeostasis	1.53 × 10^−6^	1/14 (0.071)	Catechol *O*-methyltransferase
TCA cycle	2.47 × 10^−5^	2/41 (0.049)	Malate dehydrogenase, ATP synthase alpha subunit
Glycolysis	8.28 × 10^−3^	3/40 (0.075)	Fructokinase, enolase, ATP synthase alpha subunit
Aspartate degradation II	8.35 × 10^−3^	1/14 (0.071)	Malate dehydrogenase

**ID**	**Associated Network Function**		**Score** a

1	Cellular Function and Maintenance, Energy Production and Lipid Metabolism		20
2	Amino Acid Metabolism, Small Molecule Biochemistry, Molecular Transport		2

a A score of 2 or higher indicates at least a 99% confidence of not being generated by random chance and higher scores indicate a greater confidence.
